# Evaluation of Different Bottom-up Routes for the Fabrication of Carbon Dots

**DOI:** 10.3390/nano10071316

**Published:** 2020-07-04

**Authors:** Diana M. A. Crista, Joaquim C. G. Esteves da Silva, Luís Pinto da Silva

**Affiliations:** 1 Chemistry Research Unit (CIQUP), Faculty of Sciences of University of Porto, R. Campo Alegre 697, 4169-007 Porto, Portugal; up200702319@fc.up.pt (D.M.A.C.); jcsilva@fc.up.pt (J.C.G.E.d.S.); 2 LACOMEPHI, GreenUPorto, Department of Geosciences, Environment and Territorial Planning, Faculty of Sciences of University of Porto, R. Campo Alegre 697, 4169-007 Porto, Portugal

**Keywords:** carbon dots, bottom-up synthesis, photoluminescence, life cycle assessment

## Abstract

Carbon dots (CDs) are carbon-based nanoparticles with very attractive luminescence features. Furthermore, their synthesis by bottom-up strategies is quite flexible, as tuning the reaction precursors and synthesis procedures can lead to an endless number of CDs with distinct properties and applications. However, this complex variability has made the characterization of the structural and optical properties of the nanomaterials difficult. Herein, we performed a systematic evaluation of the effect of three representative bottom-up strategies (hydrothermal, microwave-assisted, and calcination) on the properties of CDs prepared from the same precursors (citric acid and urea). Our results revealed that these synthesis routes led to nanoparticles with similar sizes, identical excitation-dependent blue-to-green emission, and similar surface-functionalization. However, we have also found that microwave and calcination strategies are more efficient towards nitrogen-doping than hydrothermal synthesis, and thus, the former routes are able to generate CDs with significantly higher fluorescence quantum yields than the latter. Furthermore, the different synthesis strategies appear to have a role in the origin of the photoluminescence of the CDs, as hydrothermal-based nanoparticles present an emission more dependent on surface states, while microwave- and calcination-based CDs present an emission with more contributions from core states. Furthermore, calcination and microwave routes are more suitable for high-yield synthesis (~27–29%), while hydrothermal synthesis present almost negligible synthesis yields (~2%). Finally, life cycle assessment (LCA) was performed to investigate the sustainability of these processes and indicated microwave synthesis as the best choice for future studies.

## 1. Introduction

Carbon dots (CDs) are quasi-spherical carbon-based nanoparticles, with a core that can be either amorphous or nanocrystalline [1,2,3,4]. The core of these nanomaterials is expected to be composed mainly of graphitic sp^2^ carbon connected by amorphous sp^3^ carbon atoms in between. The surface can present different functionalization degrees, depending on the chosen precursors and synthesis routes, and thus, different functional groups can be found (such as carboxylic acids, alcohols, and amines) [1,5].

Carbon dots belong to the broad family of carbon allotropes and related nanostructures and molecules. Carbon allotropes include diamond with sp^3^ hybridization, graphite, graphene, and carbon nanotubes with sp^2^ hybridization, fullerenes with hybridization between sp^2^ and sp^3^, as well as graphyne and carbynes with sp hybridization [6,7,8].

CDs have attracted significant attention from the research community due to their quite remarkable features, such as high photoluminescence [2,9,10], low toxicity [11], good water solubility [2,12], biocompatibility [13,14], and good physical–chemical and photochemical stability [12,15,16].

Therefore, it is not surprising that CDs have gained relevance in various fields, such as sensing [17,18,19,20], bioimaging [21], photocatalysis [10], drug delivery [22], solar cells [23], and photodynamic therapy [24].

CDs can be obtained by two main synthesis routes: top-down and bottom-up [3,25,26]. The former procedure is based on the breakdown of large graphitic materials into smaller carbon-based materials [27]. However, top-down approaches generally require harsh reaction conditions, expensive materials/equipment, and long processing times. By their turn, bottom-up routes are arguably the most widely used synthesis methods [3,25,26]. They generally consist on the pyrolysis of smaller organic molecules either in powder form (calcination) [28] or in solution via hydrothermal [25,29] or microwave-based approaches [16,30,31]. Bottom-up strategies have the advantage of being suitable for mass production, being eco-friendly, and of low cost [26].

While top-down strategies are restricted to using carbon “bulk” samples as precursors (such as graphite powders, graphene, and black carbon) [5], bottom-up procedures can use a myriad of molecular organic precursors, which range from smaller chemicals (as carbohydrates and acids) [1,3] to more heterogenous sources (as rice, coffee beans, and waste) [32,33,34,35]. Due to the structure, surface-functionalization and photoluminescence of the CDs are heavily dependent on the choice of precursors and synthesis procedures. This flexibility of bottom-up strategies allows for endless possibilities for tuning the properties of CDs and develop novel applications for them.

Among the several existent possibilities for organic precursors, citric acid (CA) is arguably the most popular one [1,3,5]. CA is readily available (being present even in several citrus fruits), cheap, nontoxic, and has a low carbonization temperature, thereby being the choice for several researchers. However, synthesis using just CA generally leads to CDs with low photoluminescence [36]. Therefore, researchers typically add also precursors containing appropriate heteroatoms (such as N, S, and P) to achieve heteroatom-doping of CDs [37], which generally enhances the fluorescence quantum yield (QY_FL_) [38,39,40] of the CDs. Among these, N-doping is the most widely used for enhancing the QY_FL_ of CDs. N-doping is achieved by adding a nitrogen-containing small organic molecule as a nitrogen source to the carbon precursor in which urea [16,41] and ethylenediamine (EDA) [42,43] are common options.

The high flexibility of bottom-up strategies (both in terms of precursors and synthesis routes) has led to an explosion in the number of studies focusing on CDs, which resulted in countless CDs with different properties and/or applications. However, the large number of possible variables has made it difficult to understand the role of either the chosen precursors or the employed synthesis routes on the properties of the obtained CDs. Thus, at this moment it is not really possible to develop new CDs in a rational and target-oriented manner.

Moreover, significant efforts to determine the role of precursors in the structure, surface-functionalization, and photoluminescence of CDs have recently been made [1,5]. The effect exerted by the employed bottom-up route is still poorly understood due to the lack of systematic studies. Herein, in this study we have focused on evaluating the effect exerted by three representative bottom-up routes (hydrothermal, microwave-assisted, and calcination synthesis) on the properties of CDs obtained using the same precursors (CA and urea as carbon and nitrogen sources, respectively). To this end, the CDs were characterized by fluorescence, UV–Vis, and X-ray photoelectron (XPS) spectroscopy, as well as dynamic light scattering (DLS) and atomic force microscopy (AFM). A life cycle assessment (LCA) approach was also used to evaluate the sustainability and environmental impacts of each synthesis route [44,45,46].

## 2. Materials and Methods

### 2.1. Fabrication

The three types of studied CDs were prepared from the same set of precursors (urea and citric acid, CA), which were purchased from Sigma-Aldrich (St. Louis, MO, USA), via different bottom-up routes: hydrothermal-, microwave-, and calcination-assisted synthesis. The same amount of CA (0.75 g) and urea (0.25 g) was used in all syntheses. For the hydrothermal-assisted synthesis, CA and urea were mixed in 5 mL of deionized water. The reaction mixture was then placed in a Teflon-lined reactor, which was encased in a stainless-steel shell, and was heated at 200 °C for 2 h in an oven. In microwave-assisted synthesis, CA and urea were also mixed in 5 mL of deionized water. The reaction mixture solution was placed in a glass beaker and was subsequently subjected to microwave irradiation (700 W in a domestic microwave) for 5 min. Finally, calcination-assisted synthesis consisted on the one-pot thermal heating of a CA and urea powder mixture during 2 h in an oven at 200 °C placed in a glace petri box.

The synthesized CDs were subsequently suspended in water (5 mL) and purified by centrifugation (10 min at 12,000 rpm) to eliminate suspended impurities. The samples were purified by dialysis (Float-A-Lyzer^®^G2 Dialysis Device SPECTRUM^®^ (New Brunswick, NJ, USA) (molecular weight cut-off of 500 Da)) for 24 h.

### 2.2. Characterization

Fluorescence analysis was measured in a 10 mm fluorescence quartz cell by using a Horiba Jobin Yvon Fluoromax spectrofluorimeter (Madrid, Spain) using 5 nm slit widths. AFM analysis was carried out using a Veeco (Plainview, NY, USA) Metrology Multimode/Nanoscope IVA by tapping mode, using a Bruker (Billerica, MD, USA) silicon probe (model TESP-SS, resonant frequency 320 kHz, nominal force constant 42 N/m, estimated tip radius 2 nm). The Zeta Potential was measured by using a particle analyzer Anton Paar Litesizer^TM^ 500 (Graz, Austria) and a polycarbonate Omega Cuvette (Ref. 155765). X-ray photoelectron spectroscopy (XPS) analysis was recorded with a Fi Kratos Axis Ultra HAS-VISION (Manchester, UK), using a monochromatic Al-Kα radiation (15 kV, 90 W). The samples were deposited in a silica plate. Spectra were analyzed by using the CasaXPS software (Teignmouth, UK).

### 2.3. Fluorescence Quantum Yield Calculation

Fluorescence quantum yield (QY_FL_) was calculated with a standard procedure, based on the comparison of the integrated luminescence intensities and absorbance values of the synthesized CDs with a reference (which depends on the emission wavelength of the sample) quinine sulfate with the following equation:

QY_FL_^Sample^ = QY_FL_^Quinine Sulphate^ x (Grad_Sample_/Grad_Quinine Sulphate_) x (×^2^_Sample_/×^2^_Quinine Sulphate_) (1)

where Grad is the gradient from the plot of integrated fluorescence intensity versus absorbance and η is the refractive index [47]. Quinine sulfate was chosen as a reference fluorophore of known quantum yield (QY_FL_ = 0.54) [48]. Absorbance measurements were made with a UNICAM Helios Gamma, using standard quartz cells.

### 2.4. Scope and System Boundaries

This cradle-to-gate study aims to quantify and compare the potential environmental impacts as well as characterize and evaluate the differences found in the three different methods for the synthesis of CDs: hydrothermal, calcination, and microwave-assisted synthesis. In this research we will study the laboratory-scale manufacturing stage of the target nanoparticles and consider the direct emissions from CDs production and the indirect impacts associated with upstream resource extraction and energy generation. Three different synthetic strategies were used. CA and urea were used as carbon and nitrogen source, respectively. The environmental impacts were compared first by using a weight-based functional unit of 1 Kg of CDs. These impacts were also normalized by the QY_FL_ of the different CDs. This is needed because weight-based functional units do not consider functional benefits for which they were engineered for. The QY_FL_ was chosen as the normalization factor because, while CDs have many different applications, a high QY_FL_ is generally a very important property in most of these applications.

### 2.5. Life Cycle Inventory Data

The environmental impacts related to these three different synthesis routes were evaluated based on inventory data from laboratory-scale synthesis procedures found in the Ecoinvent^®^ 3.5 database. The foreground system of the synthesis procedure consists of chemicals used as raw material and electricity used in the fabrication process and purification steps (oven, microwave, heating plate, centrifuge, stirring plate). The different processes and chemicals included in this study were modeled with the following data presented in the Ecoinvent^®^ 3.5 database (GLO standing for global, RER for regional market for Europe, and PT for Portugal):

- Citric acid {GLO}|Market;
- Urea, as N {GLO}|Market;
- Water, deionized, from tap water, at user {Europe without Switzerland}|Market;
- Electricity, medium voltage|Market;
- Chemical Waste, unspecified.

The chemical amount used is described in Table S1 and was rescaled to the amount needed to produce 1 Kg of CDs. The dataset used for electricity describes the available electricity data on the medium voltage level in Portugal in 2014, as described in the Ecoinvent^®^ 3.5 database. The electricity consumption considered here combines the electricity required for using either a domestic microwave (microwave-assisted synthesis) or oven (hydrothermal or calcination-based synthesis) with electricity used during the purification steps (centrifugation and dialysis). Microwave-assisted synthesis was made in an Electronia domestic microwave (model P70B17L-DE) with a power consumption of 700 W. The hydrothermal-assisted and calcination-based syntheses were made in a Furnace 47900 from Thermolyne with a power consumption of 1000 W. Centrifugation was made with a power consumption of 180 W. Stirring was done using a Jenway (Staffordshire, UK) model 1000 stirrer, with 500 W maximum power consumption.

### 2.6. Environmental Impact Assessment

This study is based on the cradle-to-gate approach, from the production of precursor materials to the fabrication of CDs. Environmental impacts were modeled using the ReCiPe 2016 V1.03 endpoint method, Hierarchist version, which evaluates three categories of potential impacts: Human Health, Ecosystems, and Resources. In the Human Health subsection the following is evaluated: global warming–human health (GW-HH), stratospheric ozone depletion (SO), ionization radiation (IR), ozone formation–human health (OF-HH), fine particulate matter formation (FPM), human carcinogenic toxicity (HC), human non-carcinogenic toxicity (HNC), and water consumption–human health (WC-HH). Ecosystems potential impacts evaluate the following: global warming–terrestrial ecosystems (GW-TE), global warming–freshwater ecosystems (GW-FE), ozone formation–terrestrial ecosystems (OF-TE), terrestrial acidification (TA), freshwater eutrophication (FE), marine eutrophication (ME), terrestrial ecotoxicity (TET), freshwater ecotoxicity (FET), marine ecotoxicity (MET), land use (LU), water consumption–terrestrial ecosystem (WC-TE), and water consumption–aquatic ecosystems (WC-AE). In the Resources subsection the following is evaluated: mineral resource scarcity (MR), fossil resource scarcity (FR). LCA study was performed using the SimaPro 9.0.0.48 software (Amersfoort, The Netherlands).

## 3. Results and Discussion

### 3.1. Synthesis

**Figure 1:**
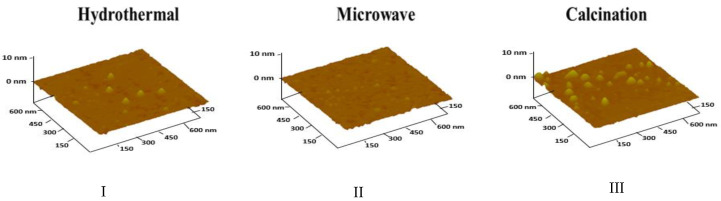
Atomic force microscopy (AFM) images for (**I**) hydrothermal-carbon dots (CDs), (**II**) microwave-CDs, and (**III**) calcination-CDs.

AFM measurements (Figure 1) were performed to determine the size of the three types of obtained CDs, and to ensure that they were indeed nanosized. AFM results provided the following average nanoparticles sizes; 6.9 ± 2.0 nm for hydrothermal-CDs, 7.3 ± 1.7 nm for microwave-CDs, and 6.1 ± 1.7 nm for calcination-CDs.

The synthesis yield (weight-by-weight, in %) (Table 1) was calculated considering the amount of final powder (after purification step) and initial precursors. The reaction yields obtained were satisfactory in the case of microwave- and calcination-assisted syntheses with 31.6% and 26.1%, respectively. In fact, the obtained synthesis yields for those two CDs are in line with values obtained in studies aiming to high-yield synthesis of CDs [49]. Unfortunately, the synthesis yield for hydrothermal-CDs was quite low (1.5%), which indicates that this type of bottom-up route is not appropriated for high-yield synthesis.

### 3.2. Surface Characterization

The surface of the three types of CDs was characterized by XPS spectroscopy (Figures S1–S3), with the XPS atomic composition (at %) of each sample being presented in Table 2. The data showed that all CDs are composed by C (~60–62%), N (~9–13%), and O (~24–28%). Interestingly, we can see that the studied bottom-up routes can lead to different degrees of N- and O-incorporation in the surface of CDs. Namely, hydrothermal synthesis led to both the highest amount of O (28.8%), which was achieved with the lowest amount of N (9.1%). By its turn, microwave-assisted synthesis appears to be the most well-balanced route in this respect, with relatively high incorporation of both N (13.1%) and O (26.9%). These findings are in line with literature, as a recent study performed by our group also revealed that microwave-assisted synthesis led to higher N-incorporation than a hydrothermal route for the same set of precursors, at the expense of O-incorporation [25]. On the contrary, calcination achieved the highest incorporation of N (13.5%) at the expense of O (24.7%), as this type of synthesis was responsible for the lowest amount of the latter heteroatom.

Functional groups present at the surface of the CDs were identified by performing a detailed scan for internal levels of C 1s, O 1s, and N 1s (Figures S1–S3). C 1s spectra could be split into three peaks (Figure S1) in the case of microwave and calcination synthesis: at binding energies of ~285 eV (attributed to C-C/C-H groups) [50], ~286 eV (C-O/C-N groups) [51], and ~288 eV (O-C = O) [52]. In the case of hydrothermal synthesis, four peaks were found instead: at binding energies of ~285 eV (attributed to C-C/C-H groups), ~286 eV (C-O/C-N groups), ~287 eV (C = O groups), and ~288 eV (O-C = O). Thus, all produced CDs presented a higher contribution from C-C/C-H groups (~56–60%), followed by O-C = O groups (~20–36%) and C-O/C-N groups (~9–12%). For hydrothermal-CDs, we also observed a contribution from C = O groups (~9%). O 1s could be split into two peaks at a binding energy of ~531 eV (C = O groups) and ~532 eV (C-O groups) [52]. The three synthesized CDs present a similar composition of almost 50% from both groups: C-O groups (~50.01%) and C = O groups (~49.99%). Finally, N 1 s could also be split into two peaks at binding energies of ~400 eV (amine/amide groups) and ~401 eV (protonated amides). All three samples show the same contribution from protonated amines (~49.98%) and amine/amide groups (~49.98%). Thus, the three different routes appear to generate the same type of functional groups in the surface of the CDs. The main difference is then the apparently higher presence of C = O groups in the surface of hydrothermal-CDs, which is in line with their higher content of both C (62.0%) and O (28.8%).

The zeta-potential of all CDs (Table 1) was measured by DLS in aqueous solutions: −0.5 mV for hydrothermal-CDs, 0.0 mV for microwave-CDs, and −0.1 mV for calcination-CDs. These results indicate that all three CDs present very a similar charge and can be considered virtually as neutral particles. This similarity is not surprising as XPS analysis revealed similar types of surface functional groups. The slightly higher negative charge for hydrothermal-CDs is also not unexpected, given the lower amount of N (9.1%) in comparison to the other two CDs (13.1–13.5%), which can lead to a lower amount of cationic functional groups (such as protonated amides). Nevertheless, the almost neutral values for all CDs indicate that these nanoparticles are prone to aggregation in solution.

### 3.3. Fluorescent Characterization

**Figure 2:**
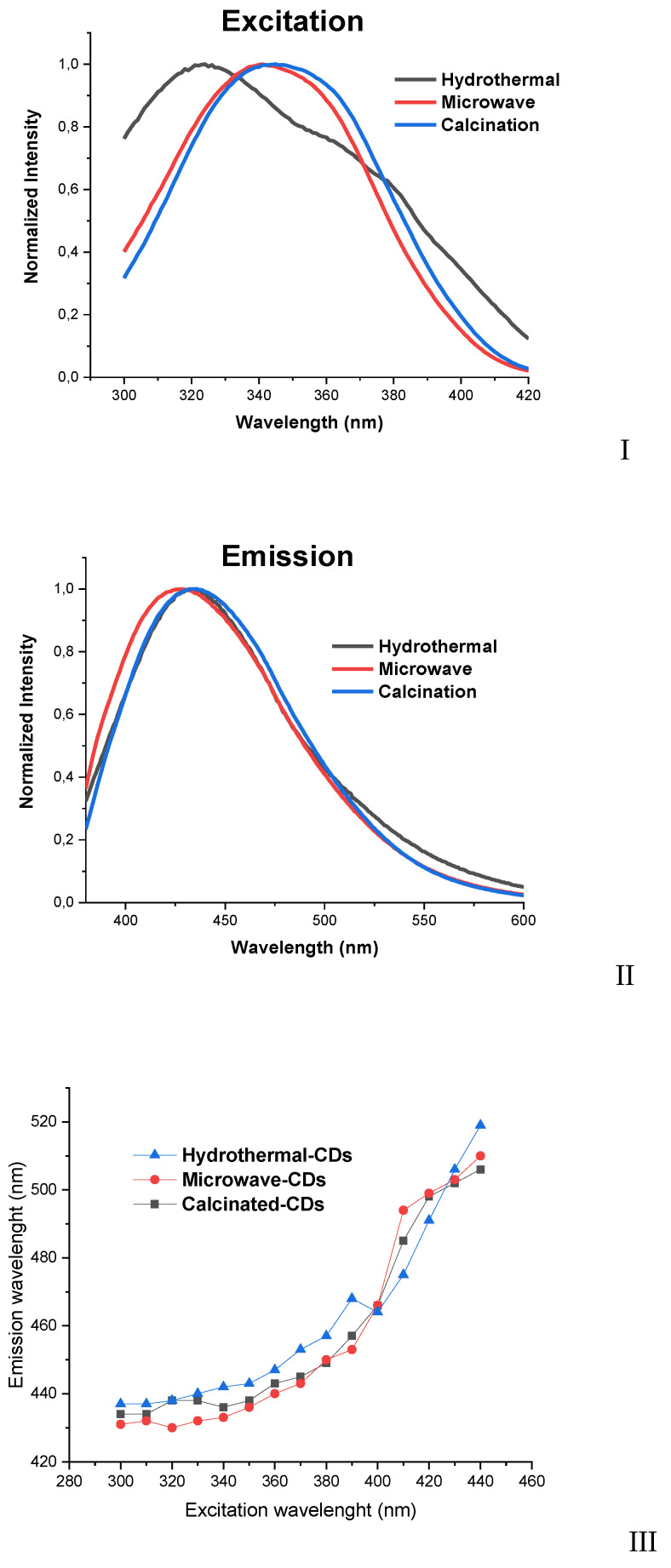
(**I**) Excitation- and (**II**) emission-spectra in aqueous solution from hydrothermal-, microwave-, and calcinated-CDs at 320, 340, and 330 nm excitation wavelength, respectively, and (**III**) emission wavelength (nm) as a function of the excitation wavelength (nm).

The absorption (Figure S4), excitation (Figure 2), and emission (Figure 2) spectra of the three CDs were obtained in aqueous solution. The absorption spectra for the CDs are very similar and present a main band at ~350 nm and a shoulder at ~245 nm, which can be attributed to n → π* and π → π* transitions [16]. In terms of fluorescence, the three bottom-up routes do not appear to induce significant differences, as all CDs present similar blue emission (433–438 nm) and UV excitation (320–340 nm). Nevertheless, it can be seen that hydrothermal-CDs present a blue-shifted excitation when comparing with other CDs. This can be attributed to the lower N-doping of the former CDs (Table 1), as N-doping is known to red-shift the excitation of CDs [53].

As shown in Figure 2II, all samples present an excitation-dependent emission which is a typical characteristic of CDs [54,55]. As we said before, all samples exhibit a blue fluorescence and when we increase the excitation wavelength from 300 to 440 nm, we observe a red-shift of ~80 nm towards green emission.

While the position of the fluorescence peak is identical for the three CDs, their intensity is not. Namely, both microwave-CDs (25.1%) and calcination-CDs (29.3%) present significantly higher QY_FL_ than hydrothermal-CDs (3.7%). Thus, hydrothermal synthesis is at a disadvantage when we compare it with microwave and calcination synthesis. As for the reason of the lower QY_FL_ for hydrothermal-CDs, it should be noted that N-doping strategies have been demonstrated to generate high QY_FL_ [56,57]. In fact, XPS analysis (Table 2) has demonstrated that hydrothermal synthesis leads to a lower degree of N-incorporation into the surface of CDs than the other two bottom-up routes, which may explain their lower QY_FL_. Furthermore, previous research by Maser et al. showed that the abundance of amide groups in the surface of CDs can lead to higher photoluminescence by favoring a more rigid structure, due to photoinduced charge transfer between amide and carboxylic groups [58]. Therefore, it is possible that hydrothermal-CDs possess lower amide groups compared to the other CDs due to a lower N-content (Table 2), leading to lower QY_FL_. In summary, our data revealed that while the three studied bottom-up routes lead to identical fluorescence wavelengths (probably due to generating similar surface functional groups), they can lead to significantly different QY_FL_ values, which can be attributed to their different efficiencies regarding heteroatom (O and N) doping.

**Figure 3:**
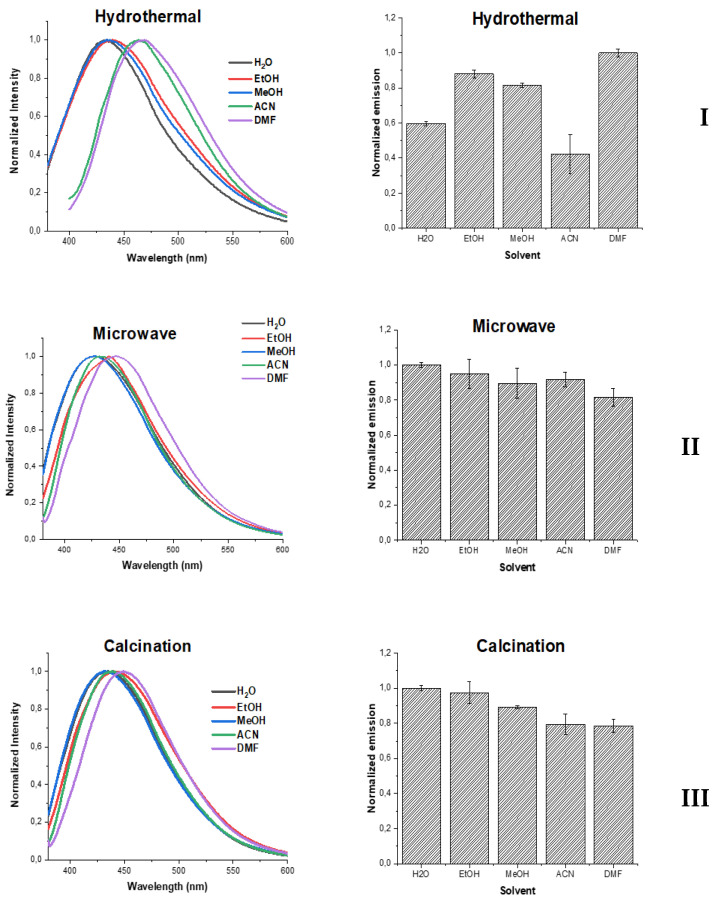
Normalized emission spectra for the three CDs: (**I**) hydrothermal-CDs, (**II**) microwave-CDs, and (**III**) calcination-CDs.

Despite extensive research, the origin of photoluminescence of CDs is still a matter of debate. A particular focus of research in recent years is to determine the role and relevance of core and surface states to the emission of these nanomaterials [59,60,61]. Therefore, we have also analyzed the effect exerted by the external environment to the fluorescence of the three studied CDs. More specifically, we have measured their fluorescence spectra in different protic and aprotic solvents: water, ethanol (EtOH), methanol (MeOH), acetonitrile (ACN), and dimethylformamide (DMF). The obtained results can be observed in Figure 3.

Interestingly, the solvent affected the three CDs in different ways. Hydrothermal-CDs were the most affected nanoparticles as their fluorescent intensity varied widely with the solvent. Furthermore, the emission band of hydrothermal-CDs appears to be particularly influenced by hydrogen-bonding, as they presented the same emission maxima (~440) in the three protic solvents (water, ethanol, and methanol). However, their emission maxima underwent a ~35 nm red-shift (to ~475 nm) in the two studied aprotic solvents (DMF and acetonitrile). These results indicate that the fluorescent moieties of hydrothermal-CDs should be exposed to the external environment, and thus, their emission should have a relevant contribution from surface states. On the contrary, the solvent has limited influence on the emission of both microwave-based and calcination-CDs. More specifically, the solvent does not significantly affect their emission bands, with the maximum red-shift being just ~10 nm in DMF. The changes in fluorescence intensity are also similar between microwave-based and calcination-CDs, and less relevant for hydrothermal-CDs. Thus, we can conclude that the fluorescence moieties of the former CDs are shielded from the external environment, and thus, their emission should originate more from core states with more limited influence from surface states.

We have also analyzed the effect of the methyl viologen (a known electron acceptor) on the emission of the CDs (Figure S5). Methyl viologen did not induce any appreciable effect on the emission of the three CDs, which indicates that they are not electron-donors.

### 3.4. Comparative LCA Study

In this section, the three different synthesis routes were first evaluated individually to analyze their impact contributions in each category using a weight-based functional unit to produce 1 Kg of CDs. As in line with previous studies from our group [25,31], the use of CA accounts for most environmental impacts to all categories (Figures S5–S7). Urea is clearly the second highest contributor to the different categories for hydrothermal synthesis, especially for the Resources category (Figure S6). On the contrary, the environmental impacts of electricity consumption are relatively negligible for hydrothermal synthesis (Figure S6). The profile of environmental impacts for calcination and microwave synthesis is somewhat different (Figures S7 and S8). Namely, for these two synthesis routes, both urea and electricity consumption present more relevant contributions to the different categories, despite CA still being the highest one. Moreover, electricity is now the second highest contributor for the global categories of Human Health and Ecosystems (Figures S7 and S8), for both calcination and microwave synthesis. Water consumption presents negligible contributions for both hydrothermal and microwave synthesis, while being non-existent for the calcination route.

**Figure 4:**
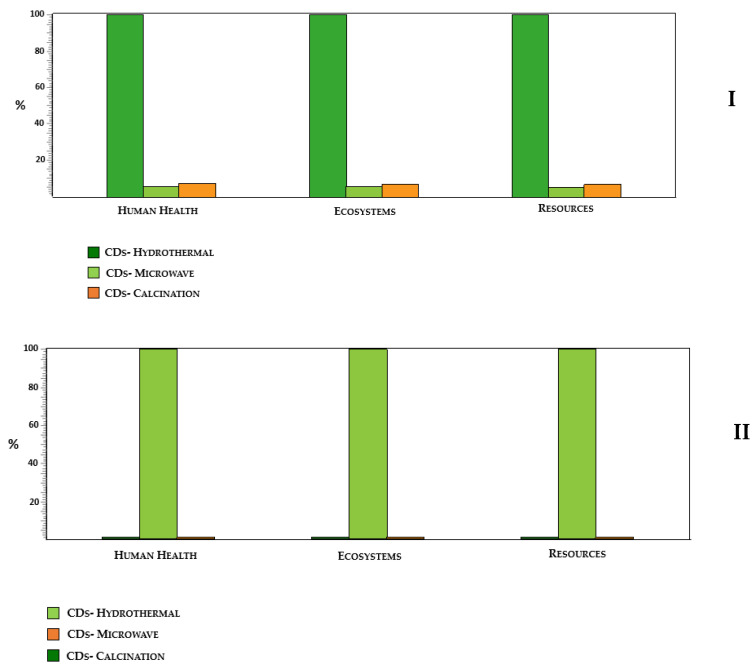
Comparative damage assessment for all three syntheses using (**I**) reaction yield and (**II**) quantum yield (QY) unit.

When we compare the relative impacts of the three synthesis routes, we can clearly see that the hydrothermal synthesis has the highest contribution for the environmental impact when compared to the other two syntheses (Figure S9 and Figure 4I. While microwave and calcination approaches provide similar impacts, we can identify the latter as the synthesis route with less environmental impacts, when we consider a weight-based functional unit. These results are easily explained by the synthesis yields (Table 1) of these synthetic strategies in which the yield for hydrothermal synthesis is almost negligible while the other two approaches provide more relevant yields (with microwave-assisted synthesis being the one with higher yield). Thus, microwave synthesis appears to be a better option due to its higher reaction yield, and consequently, lower need of chemicals per 1 Kg of CD production.

The sustainability of the three synthesis routes was also analyzed by rescaling the previous results with respect to the QY_FL_ of the three CDs (Table 3). This re-scaling was performed by considering the highest QY_FL_ (that of calcination-CDs) as the reference QY_FL_ (QY_FL_^REF^). The QY_FL_-normalized functional unit for each CDs was calculated as QY_FL_^REF^/QY_FL_ (Table 3).

This re-scaling did not affect the relative position of hydrothermal-CDs (Figure S9II and Figure 4II), as these CDs present both the lowest synthesis yield and QY_FL_ (Table 1). Interestingly, if we just compare just the environmental profiles of microwave-based and calcination-CDs (Figure S10), the latter nanoparticles are the ones that present the lowest environmental impacts, despite the former being the ones to present the highest QY_FL_. This indicates that the better performance of calcination-CDs in terms of QY_FL_ is not enough to offset their higher requirement for initial precursors. In conclusion, our LCA analysis revealed that microwave-assisted synthesis should be the preferred bottom-up route when considering both a weight- and a function-based function, while hydrothermal synthesis is clearly the worst option.

## 4. Conclusions

We have performed a systematic study of the structural and optical properties of carbon dots (CDs) prepared by three representative bottom-up strategies (hydrothermal, microwave-assisted, and calcination synthesis), from the same precursors (citric acid and urea), with the objective of understanding the effect exerted by synthesis methods on the properties of novel CDs. Our results revealed that microwave and calcination are more suitable for high-yield synthesis, with the hydrothermal strategy providing quite low synthesis yields.

Further analysis revealed that the three synthesis strategies led to nanoparticles with similar sizes, identical excitation-dependent blue-to-green emission, and similar types of surface functional groups. Interestingly, despite these similarities, both microwave and calcination strategies are more efficient for nitrogen-doping than hydrothermal synthesis, which leads to significantly more efficient emission for the former than for the latter. Moreover, the different syntheses affect the origin of their photoluminescence, as hydrothermal-CDs present emission more susceptible to the external environment, which indicates that their emission has a higher contribution from surface states. On the contrary, both microwave and calcination strategies appear to generate CDs with emission more dependent on core states than on surface states.

Finally, a life cycle assessment (LCA) analysis was performed, considering both the synthesis and fluorescence quantum yields as functional units. These results demonstrated that microwave-assisted synthesis was the strategy with the least environmental impacts in both scenarios, and thus, should be the preferred synthesis route, followed closely by calcination.

## Figures and Tables

**Table 1:** Synthesis and fluorescence quantum yields (in %) and zeta potential (in mV) data for the three CDs.

	Hydrothermal	Microwave	Calcination
**Synthesis yield/%**	1.8 ± 0.4	28.5 ± 4.5	26.9 ± 1.1
**Quantum yield/%**	3.7	25.1	29.3
**Zeta Potential/mV**	−0.5	0.0	−0.1

**Table 2:** Atomic composition (%) obtained by XPS for the three different synthesis.

	Hydrothermal-CDs	Microwave-CDs	Calcination-CDs
**C (%)**	62.0	60.0	61.9
**N (%)**	9.1	13.1	13.5
**O (%)**	28.8	26.9	24.7

**Table 3:** Fluorescence quantum yields (QY_FL_) and QY_FL_-based functional units.

Carbon Dots	QY_FL_(%)	QY_FL_-Based Functional Unit (Using Microwave Synthesis as Reference)
**Hydrothermal**	3.7	7.9
**Microwave**	25.1	1.2
**Calcination**	29.3	1.0

## Supplementary Materials

The following are available online at https://www.mdpi.com/2079-4991/10/7/1316/s1, Table S1: Inputs required for the production of 1 Kg of CDs, Figure S1: XPS C 1s spectra for calcinated-, microwave-, and hydrothermal-based CDs, Figure S2: XPS O 1s spectra for calcinated-, microwave-, and hydrothermal-based CDs, XPS N 1s spectra for calcinated-, microwave-, and hydrothermal-based CDs, Figure S4: UV–Vis spectra of the three synthesized CDs, Figure S5: Normalized intensity of different CDs in the presence of different concentrations of methyl viologen, Figure S6: Relative environmental impacts and comparative damage assessment for hydrothermal synthesis, Figure S7: Relative environmental impacts and comparative damage assessment for microwave synthesis, Figure S8: Relative environmental impacts and comparative damage assessment for calcination synthesis, Figure S9: Relative environmental impacts for all synthesis using (I) weight unit and (II) QY unit, Figure S10: (I) Relative environmental impacts and (II) comparative damage assessment for microwave and calcination synthesis using QY unit.
