# Supplementary material for: Evaluation of Different Bottom-up Routes for the Fabrication of Carbon Dots
Source: Nanomaterials (Basel). 2020 Jul 4;10(7):1316. doi: 10.3390/nano10071316 (PMC7407658; doi:10.3390/nano10071316)
Supplement: Supplementary file 1 [file nanomaterials-10-01316-s001.pdf]

Evaluation of Different Bottom-up Routes for the Fabrication of Carbon Dots

Diana M.A. Crista [1], Joaquim C.G. Esteves da Silva [1,2] and Luís Pinto da Silva [1,2,*]

[1] Chemistry Research Unit (CIQUP), Faculty of Sciences of University of Porto, R. Campo Alegre 697, 4169-007 Porto, Portugal; up200702319@fc.up.pt (D.M.A.C.); jcsilva@fc.up.pt (J.E.S.)

[2] LACOMEPHI, GreenUPorto, Department of Geosciences, Environment and Territorial Planning, Faculty of Sciences of University of Porto, R. Campo Alegre 697, 4169-007 Porto, Portugal

* Correspondence: luis.silva@fc.up.pt

Table S1. Inputs required for the production of 1 Kg of CDs.

Carbon dots	Carbon source (Kg)	Nitrogen source (Kg)	Deionized Water (Kg)	Electricity (KWh)
Hydrothermal	50	16	330	17
Microwave	2.4	0.8	20	12
Calcination	3	1	-	17

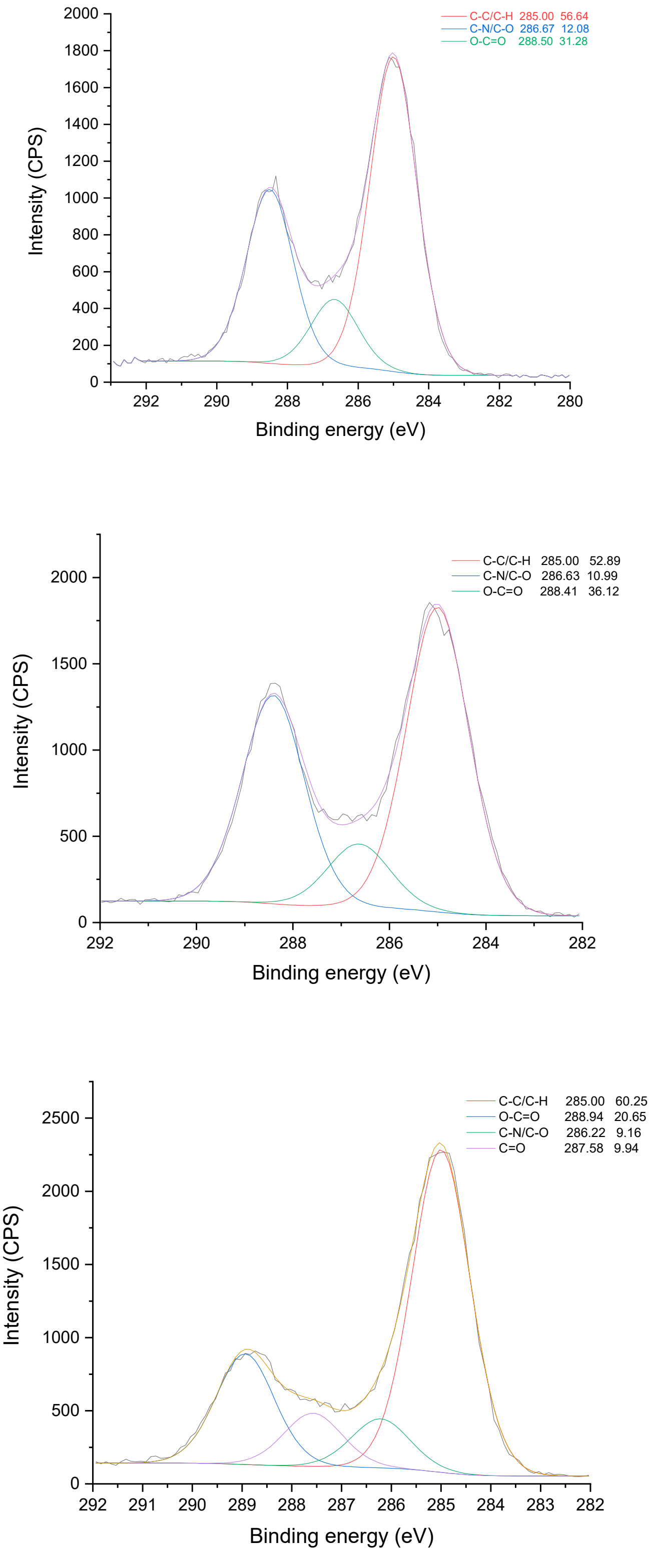

Figure S1. XPS C 1*s* spectra for calcinated- (top), microwave- (middle) and hydrothermal-based (bottom) CDs.

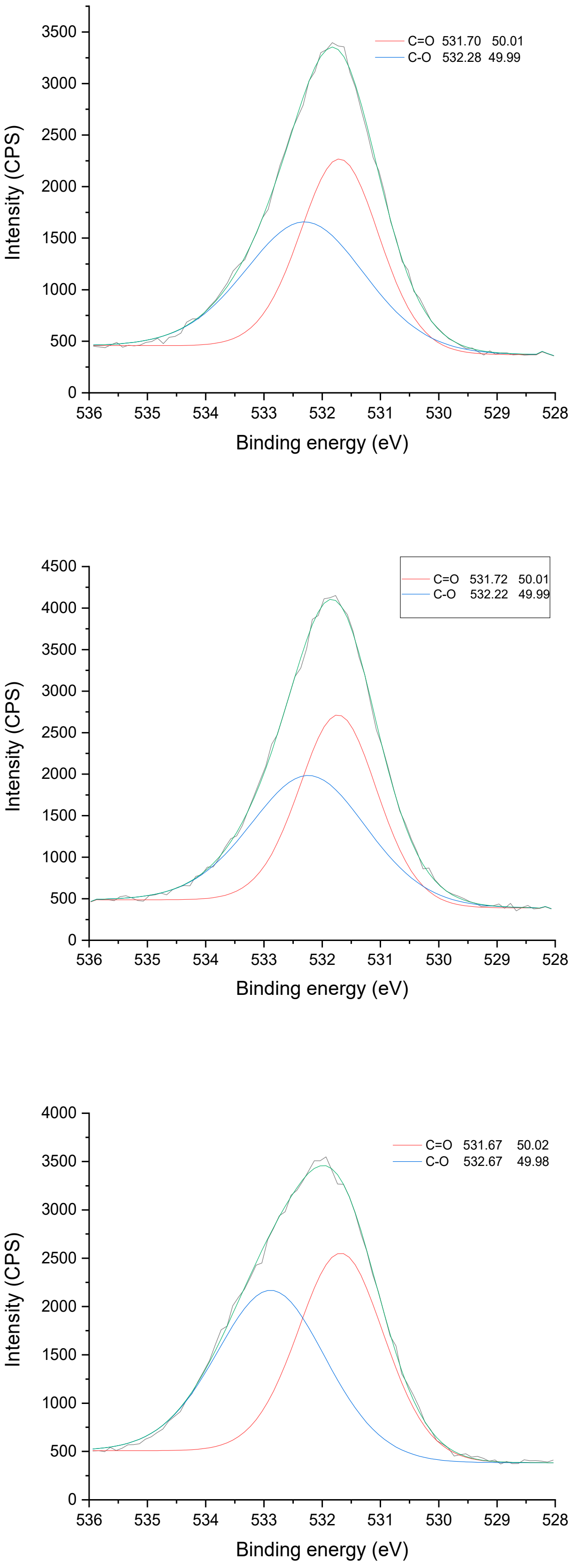

Figure S2. XPS O 1s spectra for calcinated- (top), microwave- (middle) and hydrothermal-based (bottom) CDs.

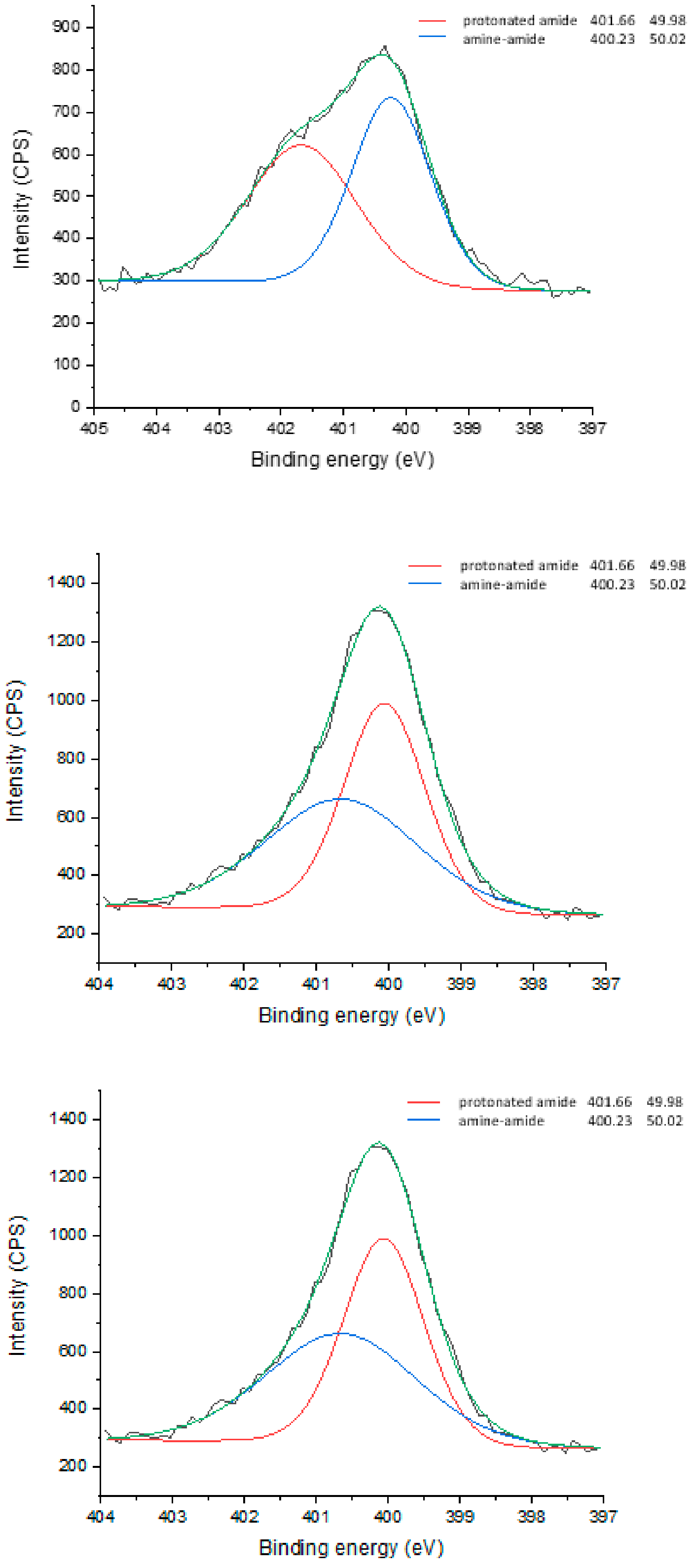

Figure S3. XPS N 1*s* spectra for calcinated- (top), microwave- (middle) and hydrothermal-based (bottom) CDs.

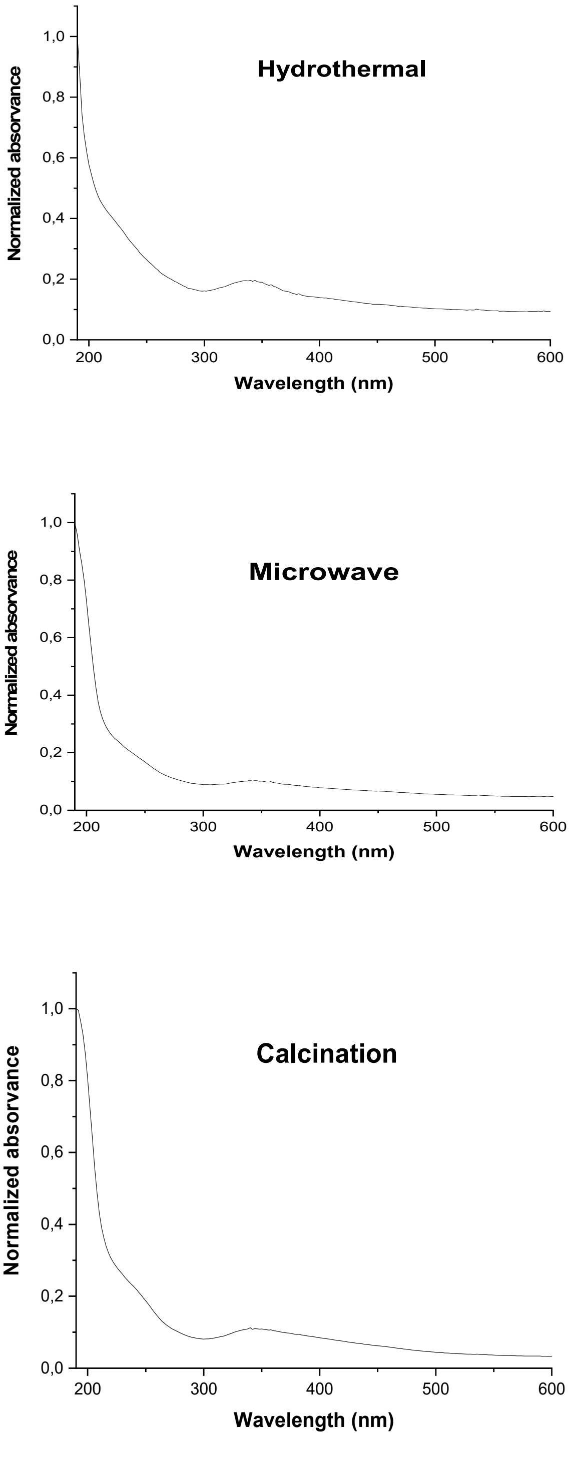

Figure S4. UV-Vis spectra of the three synthesized CDs.

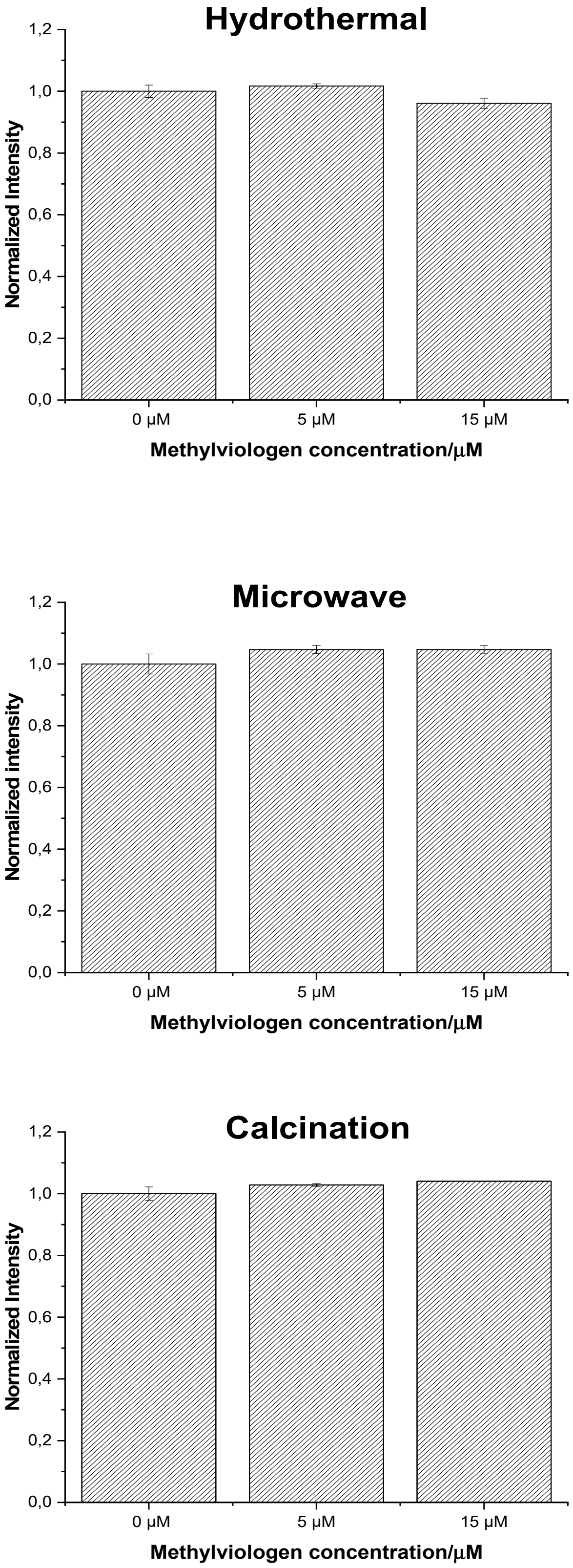

Figure S5. Normalized intensity of different CDs in the present of different concentrations of methyl viologen.

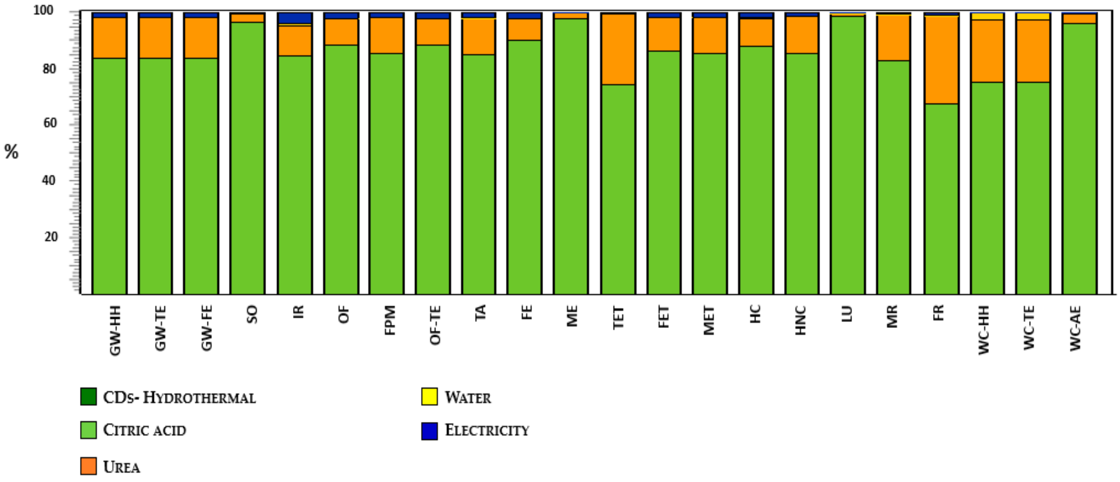

I

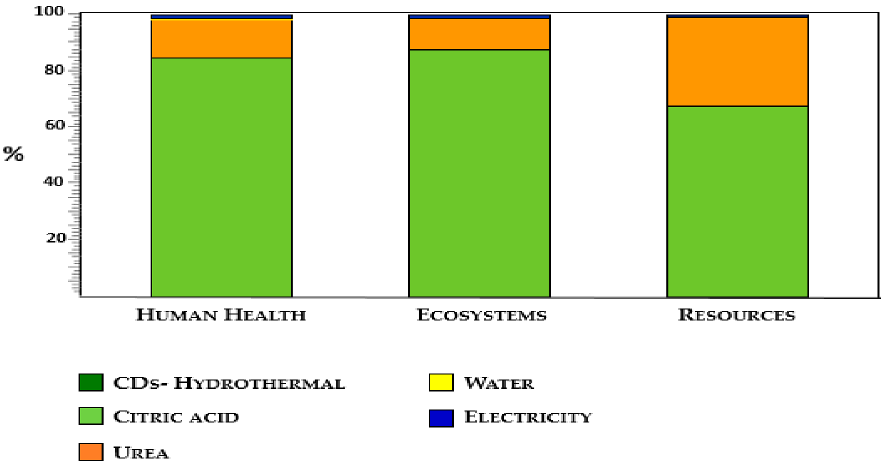

II

Figure S6. (I) Relative environmental impacts and **(II)** comparative damage assessment for hydrothermal synthesis.

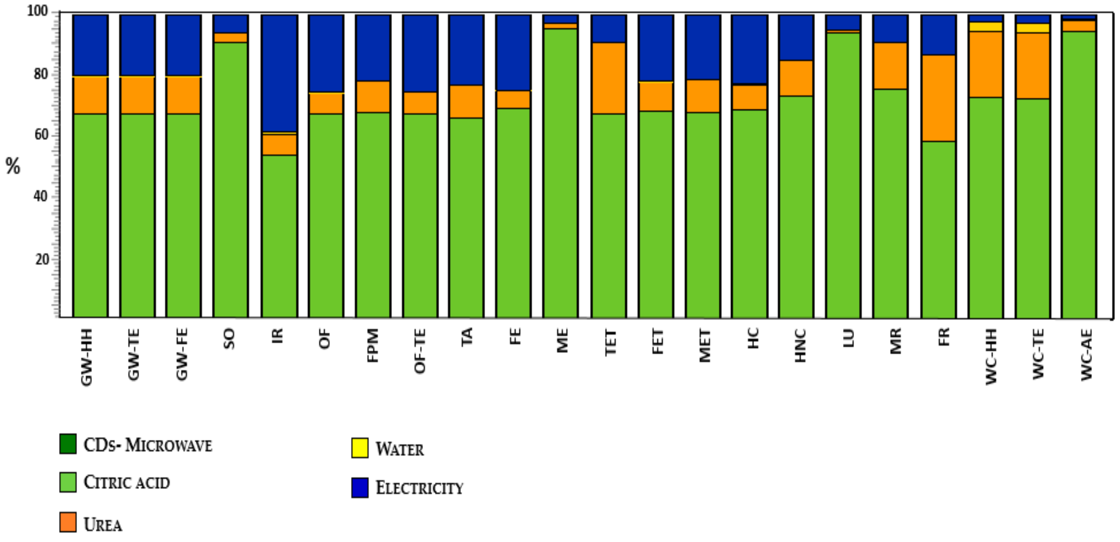

I

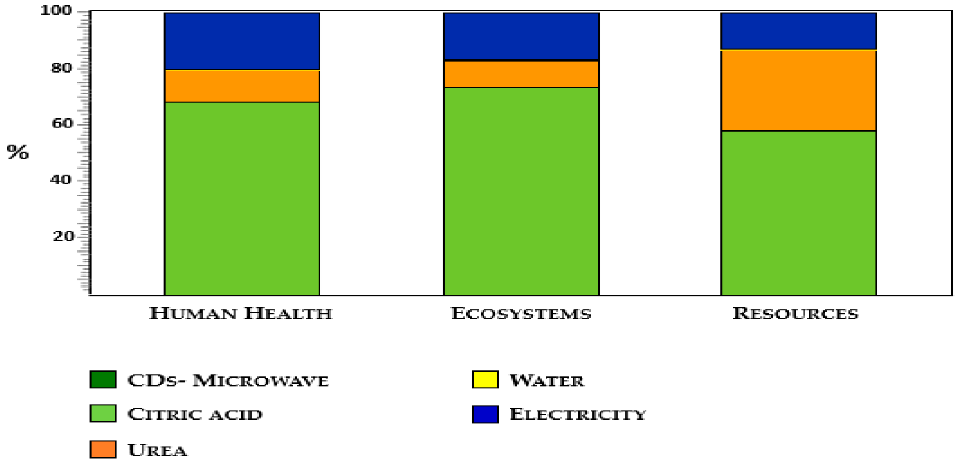

II

Figure S7. (I) Relative environmental impacts and **(II)** comparative damage assessment for microwave synthesis.

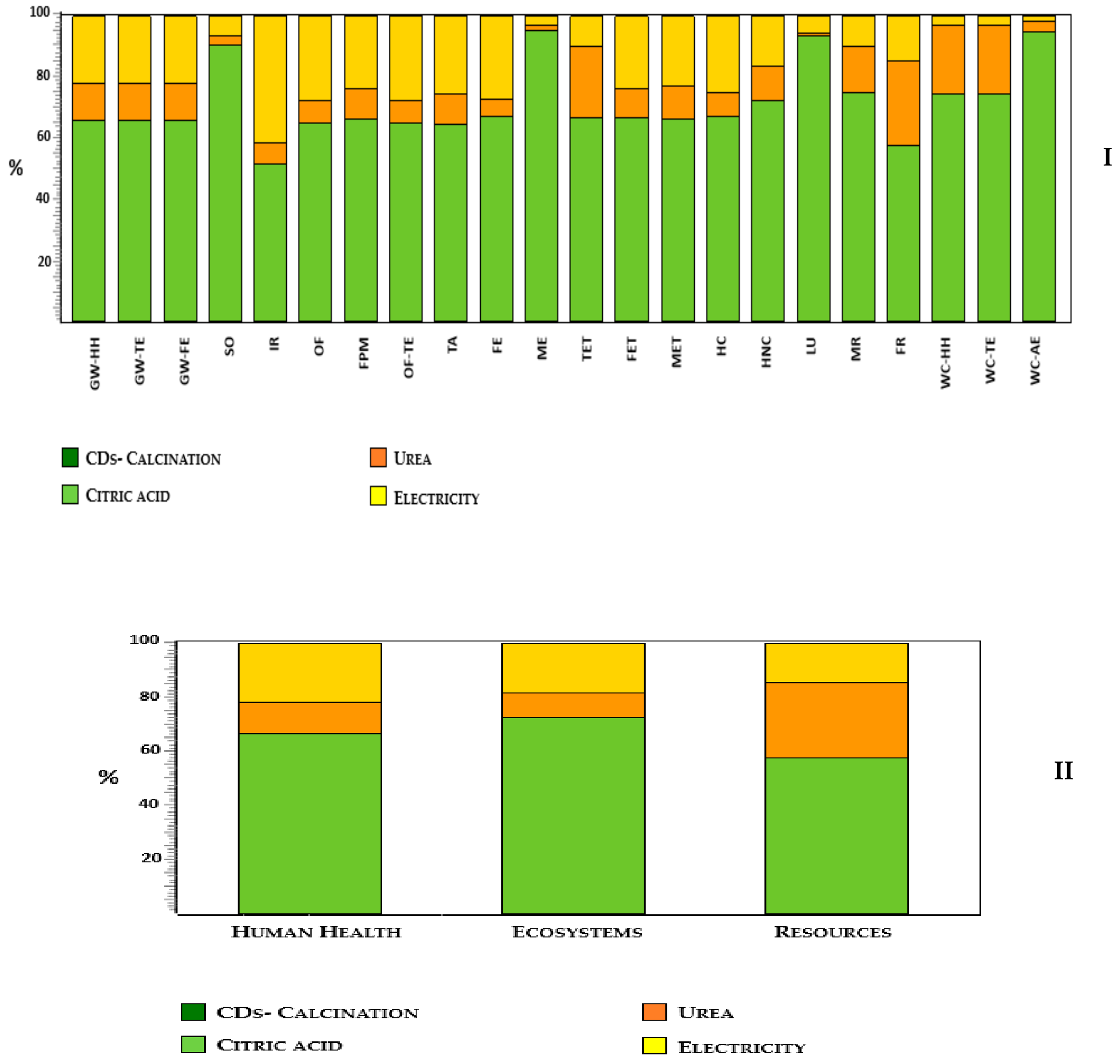

Figure S8. (I) Relative environmental impacts and **(II)** comparative damage assessment for calcination synthesis.

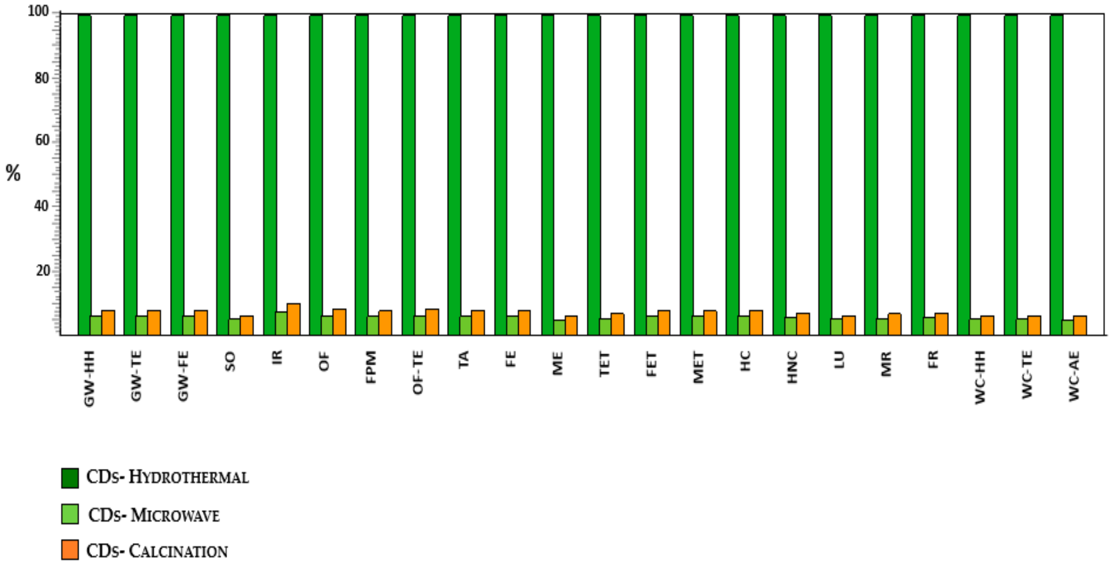

I

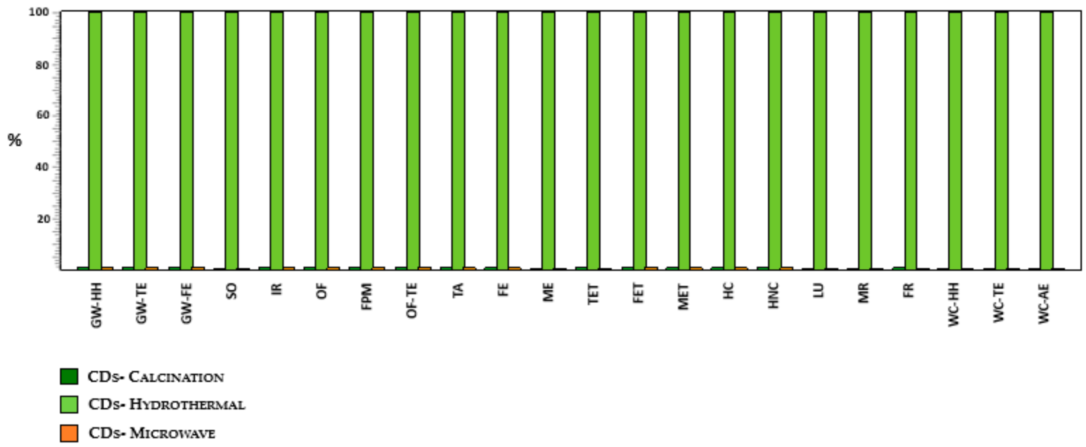

II

Figura S9. Relative environmental impacts for all synthesis using **(I)** weigth unit and **(II)** QY unit.

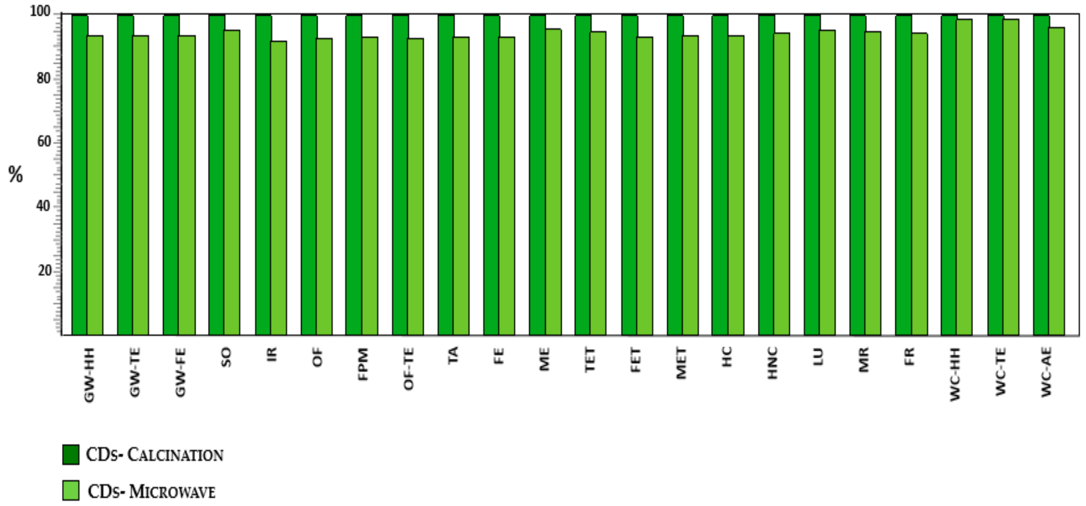

I

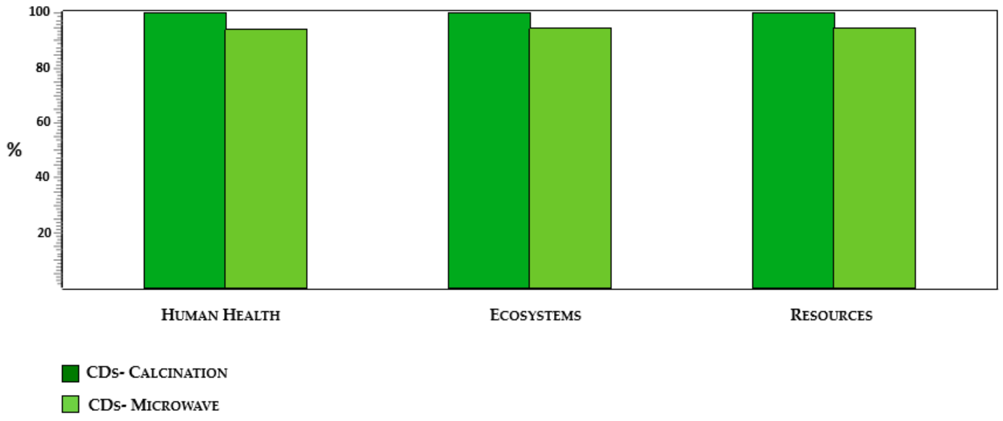

II

Figure S10. (I) Relative environmental impacts and **(II)** comparative damage assessment for Microwave- and Calcination synthesis using QY unit.
